# A High Throughput Phenotypic Screening reveals compounds that counteract premature osteogenic differentiation of HGPS iPS-derived mesenchymal stem cells

**DOI:** 10.1038/srep34798

**Published:** 2016-10-14

**Authors:** Alessandra Lo Cicero, Anne-Laure Jaskowiak, Anne-Laure Egesipe, Johana Tournois, Benjamin Brinon, Patricia R. Pitrez, Lino Ferreira, Annachiara de Sandre-Giovannoli, Nicolas Levy, Xavier Nissan

**Affiliations:** 1 CECS, I-STEM, AFM, Institute for Stem cell Therapy and Exploration of Monogenic diseases, 2 rue Henri Desbruères, 91100 Corbeil-Essonnes, France; 2 INSERM U861, I-STEM, AFM, Institute for Stem cell Therapy and Exploration of Monogenic diseases, 2 rue Henri Desbruères, 91100 Corbeil-Essonnes, France; 3 UEVE, I-STEM, AFM, Institute for Stem cell Therapy and Exploration of Monogenic diseases, 2 rue Henri Desbruères, 91100 Corbeil-Essonnes, France; 4 CNC-Center for Neurosciences and Cell Biology, University of Coimbra, Largo Marques de Pombal, 3004-517 Coimbra, Portugal; 5 Aix Marseille Université, UMR S 910: Génétique médicale et génomique fonctionnelle, Faculté de médecine Timone, Marseille, France; 6 INSERM, UMR S 910: Génétique médicale et génomique fonctionnelle, Faculté de médecine, Marseille, France

## Abstract

Hutchinson-Gilford progeria syndrome (HGPS) is a rare fatal genetic disorder that causes systemic accelerated aging in children. Thanks to the pluripotency and self-renewal properties of induced pluripotent stem cells (iPSC), HGPS iPSC-based modeling opens up the possibility of access to different relevant cell types for pharmacological approaches. In this study, 2800 small molecules were explored using high-throughput screening, looking for compounds that could potentially reduce the alkaline phosphatase activity of HGPS mesenchymal stem cells (MSCs) committed into osteogenic differentiation. Results revealed seven compounds that normalized the osteogenic differentiation process and, among these, all-trans retinoic acid and 13-cis-retinoic acid, that also decreased progerin expression. This study highlights the potential of high-throughput drug screening using HGPS iPS-derived cells, in order to find therapeutic compounds for HGPS and, potentially, for other aging-related disorders.

Hutchinson-Gilford progeria syndrome (HGPS) (OMIM #176670) is an extremely rare genetic disease that induces a premature aging in children^1^. HGPS is caused by a single base substitution (NM_170707.3, c.1284C > T) in exon 11 of *LMNA*^2^,^3^. This leads to the activation of a cryptic splicing donor site and a consequent deletion of 50 amino acids in the prelamin A protein, called progerin. Because of this deletion^4^, progerin remains farnesylated and is accumulated in the nuclear membrane, leading to a disorganization of nuclear shape and to a set of well characterized cellular dysfunctions, such as defects in DNA repair, cell proliferation, premature senescence and osteogenic differentiation (for review^5^). Recently, several studies have described that progerin expression is associated with functional impairment in mesenchymal stem cells (MSCs), reporting premature osteogenic differentiation^6^,^7^ and defective differentiation along the adipogenic^8^ and chondrogenic^9^ lineages.

Since the discovery of the molecular mechanism causing this syndrome, three drugs targeting prenylation have been used alone or in combination in the treatment of HGPS patients, lonafarnib, a farnesyltransferase inhibitor (FTI)^10^,^11^,^12^,^13^, zoledronate and pravastatin (ZoPra)^14^. Recently, several alternative approaches targeting progerin content have been described, either by decreasing its production with antisense RNA^15^ and retinoids^16^ or increasing its degradation with rapamycin^17^ and sulforaphane^18^. Interestingly, all these potential treatments were identified through hypothesis-driven experiments, based on the analysis of the molecular mechanisms involved or associated with this syndrome. Another way to identify new potential treatments is high-throughput drug screening. It has not been possible, to our knowledge, to produce the large cell banks required for such an approach from prematurely senescent HGPS primary cells. In order to overcome this problem, two complementary cellular models have been designed, one of which involves overexpressing an inducible progerin in healthy cells^19^ and the other, the reprogramming of patient cells into induced pluripotent stem cells (iPSCs)^20^.

Thanks to their pluripotency and self-renewal properties, pluripotent stem cells permit the production of an unlimited and homogeneous biological resource for testing thousands of chemical compounds^21^. Since 2011, several groups, including our own group, have demonstrated that vascular smooth muscle cells (VSMCs) and MSCs differentiated from patient iPS cells recapitulate some aspects of the syndrome, including abnormal nuclear shape architecture, progerin expression, defects in the DNA repair process and in osteogenic differentiation^22^,^23^,^24^. One advantage of this model is the reported silencing of progerin in undifferentiated iPSCs and its activation after differentiation, which makes it possible to expand the cells before their senescence^25^. Over the past two years, our group has already demonstrated that these cells can be used to decipher the functional effects of the drugs that are currently used in HGPS patients^7^ and to identify new pharmacological modulators of prelamin A farnesylation^20^. Here, we have taken advantage of this model to screen 2800 compounds for their capacity to normalize a pathological phenotype associated with this syndrome.

## Results

### High-throughput screening of 2800 compounds on osteogenic differentiation of HGPS MSCs

**Figure 1:**
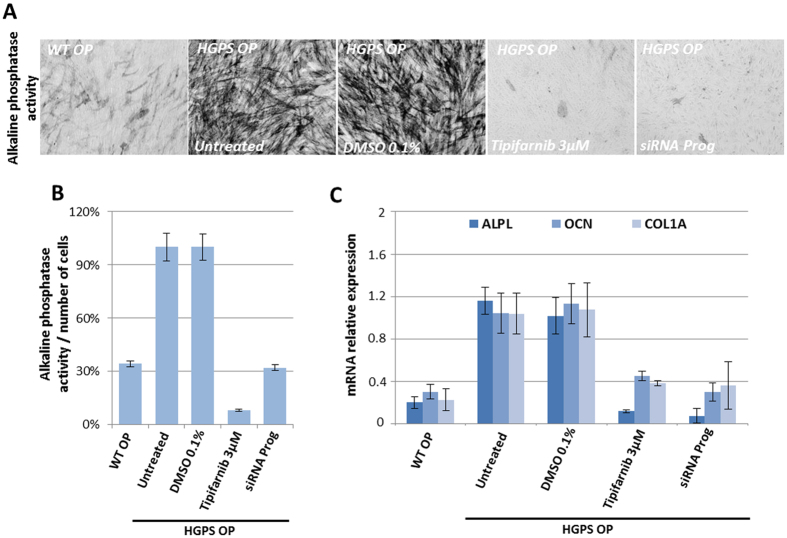
Premature osteogenic differentiation from HGPS MSCs compared with WT MSCs. (**A**) Alkaline phosphatase activity in WT and HGPS osteogenic progenitors (OP) following 7 days of differentiation in the presence of tipifarnib (3 μM) or a siRNA progerin (15 nM). (**B**) Quantification of alkaline phosphatase activity in WT and HGPS OP following 7 days of differentiation in the presence of tipifarnib (3 μM) or a siRNA progerin (15 nM). Data are normalized to cell number. (**C**) Gene expression analysis of osteogenic genes, alkaline phosphatase (ALPL), osteocalcin (OCN), collagen type 1 alpha 1 (COL1A1), in WT and HGPS osteogenic progenitors after 7 days of differentiation in the presence of tipifarnib (3 μM) or a siRNA progerin (15 nM). Data are normalized to HGPS OP treated with 0.1% DMSO.

**Figure 2:**
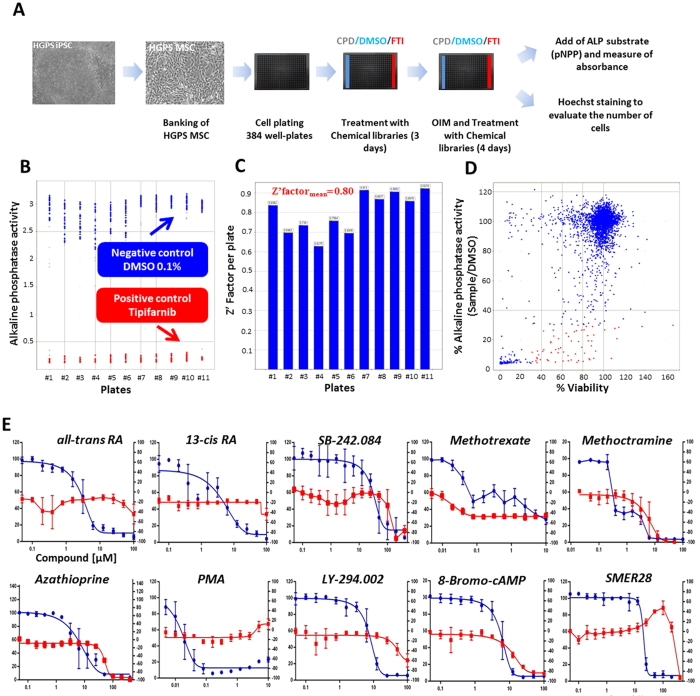
High-throughput screening of 2800 small molecules on osteogenic differentiation. (**A**) Workflow for the high-throughput screening of osteogenic modulators.CPD corresponds to compounds of the chemical library, FTI to farnesyl transferase inhibitor and OIM to ostegenic induction medium. (**B**) Validation of the screening with the alkaline phosphatase activity in HGPS osteogenic progenitors treated with the negative control, DMSO 0.1%, and the positive control, 3 μM tipifarnib, per 384-well plates. (**C**) Determination of the Z′ factor for each of the 384-well plates. (**D**) Primary screen cell-based assay for osteogenic differentiation. Dot plot representation of the effects of the 2800 compounds on alkaline phosphatase activity in %, normalized to negative control and cell viability. (**E**) Dose-response experiments for the 10 osteogenic differentiation modulators that were identified. Each chart represents cell viability (in red) and percentage of alkaline phosphatase activity (in blue). Each point represents the mean ± SD for eight replicates.

Drug screening was performed on MSCs derived from HGPS iPSCs committed to the osteoblastic lineage after four days of culture in an osteogenic induction medium. Osteogenic differentiation of MSCs was monitored through the quantification of alkaline phosphatase (ALP) activity. At this early stage of differentiation, HGPS MSCs presented increased ALP activity when compared with WT MSCs (Fig. 1A,B) and overexpressed the key osteogenic markers ALP, osteocalcin (OCN) and collagen 1A (COL1A) (Fig. 1C). The relevance of this readout was confirmed by showing that both ALP activity and osteogenic markers were rescued in the presence of the FTI tipifarnib and after progerin knockdown using a specific siRNA (Fig. 1A–C), but not in presence of regulators of osteogenesis (Sup Fig. 1). Based on these results, a high-throughput enzymatic assay was developed in order to quantify ALP activity in 384-well plates (Fig. 2A). A total of 2800 small molecules belonging to 3 different compound libraries were tested on HGPS MSCs (Sup Fig. 2A). Quantification of ALP activity was performed using spectrophotometry, by measuring the hydrolysis of p-nitrophenylphosphate (pNPP) into p-nitrophenol, a chromogenic product that absorbs at 405 nm, after four days of differentiation (Sup Fig. 2B). Cell viability was measured by counting Hoechst stained cells using an automated imaging system. DMSO 0.1% and tipifarnib (3 μM) were used as negative and positive controls, respectively (Fig. 2B). Drug screening was performed in one run with two sets of plates, the first for measuring ALP activity, the second for measuring cell viability. Quality control for the assay was ensured by the calculation of a Z′ factor between negative and positive controls that was not inferior to 0.8 (Fig. 2C). Compounds were considered as potential candidates when their effect was greater than 3 standard deviations from the mean of all tested compounds, without affecting cell viability to an extent of greater than 30% (Fig. 2D). This led to a first list of 79 hits (Sup Table 1). Retest experiments excluded 45 of these candidates because they were either toxic or judged to be false positives (Sup Fig. 2C). Efficiency and toxicity of the 34 validated compounds were evaluated in progressively higher concentrations, resulting in 10 compounds with an efficiency of greater than 50%, without cell toxicity (Fig. 2E). IC50 ranged between 25 nM–31 μM (Sup Fig. 3). Optimum concentrations were determined for each of these 10 compounds as the highest efficient concentrations acting on ALP activity without toxicity (Fig. 2E, Sup Fig. 3).

### Molecular characterization of the hit compounds

**Figure 3:**
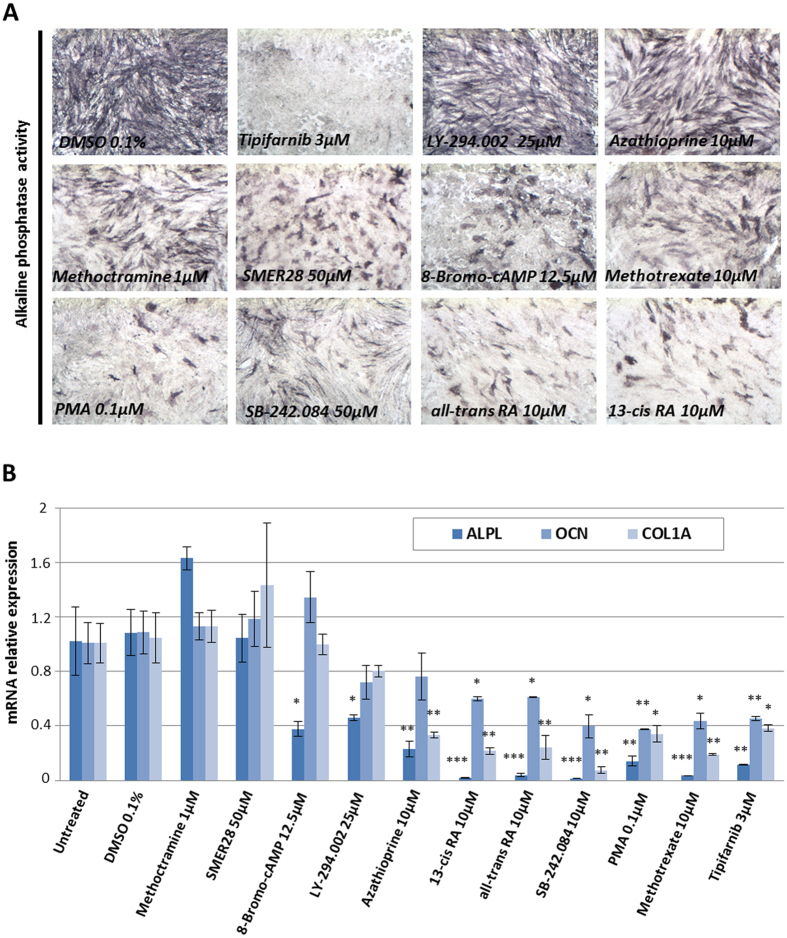
Results of the screening of osteogenic differentiation modulators. (**A**) Alkaline phosphatase activity in HGPS osteogenic progenitors following 7 days of differentiation in the presence of the 10 validated compounds. (**B**) Gene expression analysis of osteogenic genes, alkaline phosphatase (ALPL), osteocalcin (OCN), collagen type 1 alpha 1 (COL1A1), in HGPS osteogenic progenitors after 7 days of differentiation in the presence of the 10 validated compounds. Data are normalized to HGPS OP treated with 0.1% DMSO. Statistical analysis was performed with one-way analysis of variance (ANOVA), using Dunnet’s comparison test. p values < 0.05 were considered as significant (*p < 0.05, **p < 0.01, ***p < 0.001).

**Figure 4:**
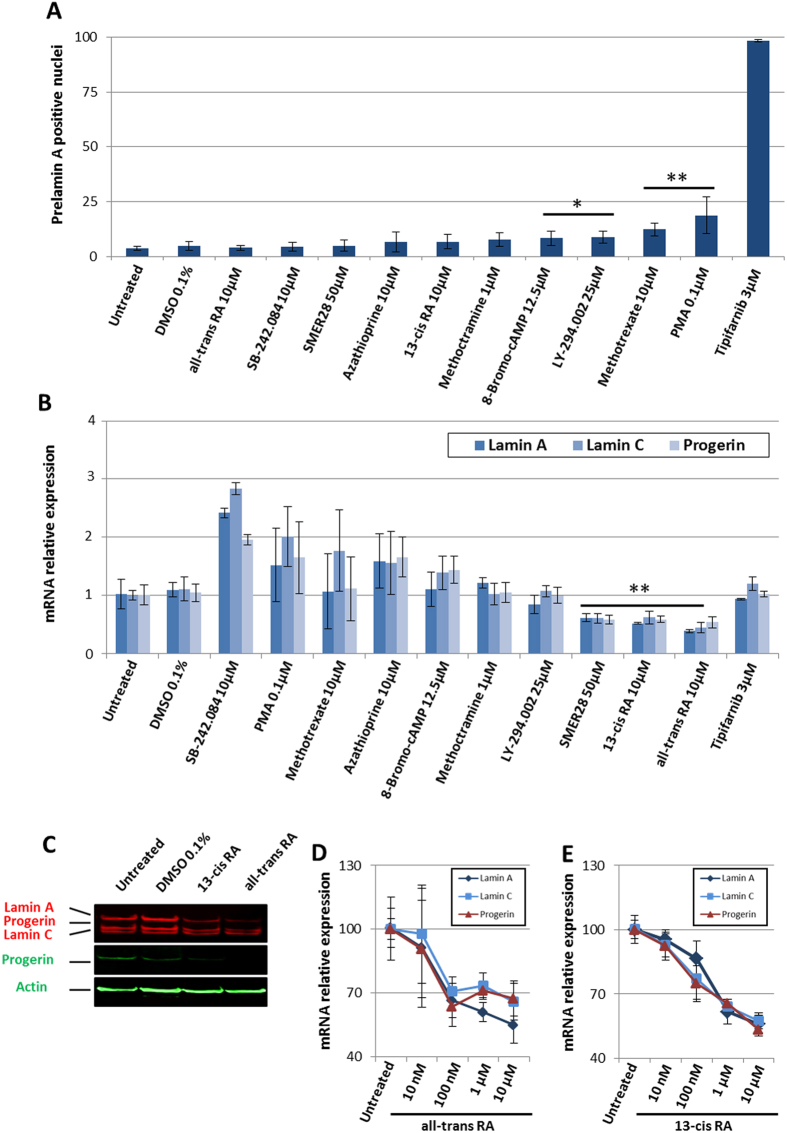
Pharmacological evaluation of the effects of the 10 validated compounds on HGPS defects. (**A**) Percentage of prelamin A positive nuclei in HGPS MSCs following 72 hours of treatment with the 10 validated compounds. Statistical analysis was performed with one-way analysis of variance (ANOVA), using Dunnet’s comparison test. Values of p values < 0.05 were considered significant (*p < 0.05, **p < 0.01, ***p < 0.001). (**B**) Gene expression analysis of lamin A/C and progerin in HGPS MSCs after 72 hours of treatment with the 10 validated compounds. Datas are normalized to untreated cells. Statistical analysis was performed with one-way analysis of variance (ANOVA), using Dunnet’s comparison test. Values of p values < 0.05 were considered significant (*p < 0.05, **p < 0.01, ***p < 0.001) (**C**) Western blot analysis of lamin A, lamin C, progerin expression in HGPS MSCs following 72 hours of treatment with retinoic acids (RA), all-trans RA (10 μM) and 13-cis (10 μM). Datas are presented as a percentage relative to untreated cells. (**D**) Dose-response analysis of lamin A, lamin C and progerin expression in HGPS MSCs after 72 hours of treatment with all-trans retinoic acid (RA). (**E**) Dose-response analysis of lamin A, lamin C and progerin expression in HGPS MSCs after 72 hours of treatment with 13-cis retinoic acid (RA). Datas are presented as a percentage relative to untreated cells.

To exclude potential false positive hits that interfered with the ALP substrate or absorbance reading, the effects of all these compounds were also confirmed using another substrate hydrolyzed by ALP, the BCIP/NBT Phosphatase Substrate (Fig. 3A). Measurements of the blue product of BCIP/NBT hydrolysis confirmed the effect of the ten hits on ALP activity (Fig. 3A). Expression of ALP, OCN and COL1A in HGPS MSCs was measured 72 h after treatment in order to discriminate between compounds that decreased ALP activity and those that regulated the osteogenic differentiation process in MSCs (Fig. 3B). Results confirmed that seven of the ten compounds significantly decreased expression of these three osteogenic markers (the exceptions were SMER28, methoctramine and 8-bromo-cAMP) (Fig. 3B). Since premature osteogenic differentiation of MSCs is sensitive to progerin content or its farnesylation, the prelamin A maturation process and progerin expression were monitored by immunostaining and qPCR, respectively. Effect of these compounds on prelamin A maturation process was assessed by measuring the percentage of HGPS MSCs presenting prelamin A nuclear staining. Tipifarnib 3 μM was used as positive control. This study revealed that four of the ten compounds partially inhibited the prelamin A farnesylation process (8-bromo-cAMP, LY-293002, methotrexate and PMA) (Fig. 4A, Sup Fig. 4A,B). Measurement of progerin was performed by RT-qPCR showing that three of these hits decreased progerin expression, SMER28, 13-cis retinoic acid (RA) and all-trans RA (Fig. 4B) (Sup Fig. 5). Finally measurement of lamin A and lamin C expression revealed that SMER28, 13-cis RA and all-trans RA did not act through regulation of alternate splicing of the *LMNA* gene, since all A-type lamin alternative transcripts decreased after treatment (Fig. 4B) (Sup Fig. 5).

### Retinoids rescue premature osteogenic differentiation by regulating progerin expression

**Figure 5:**
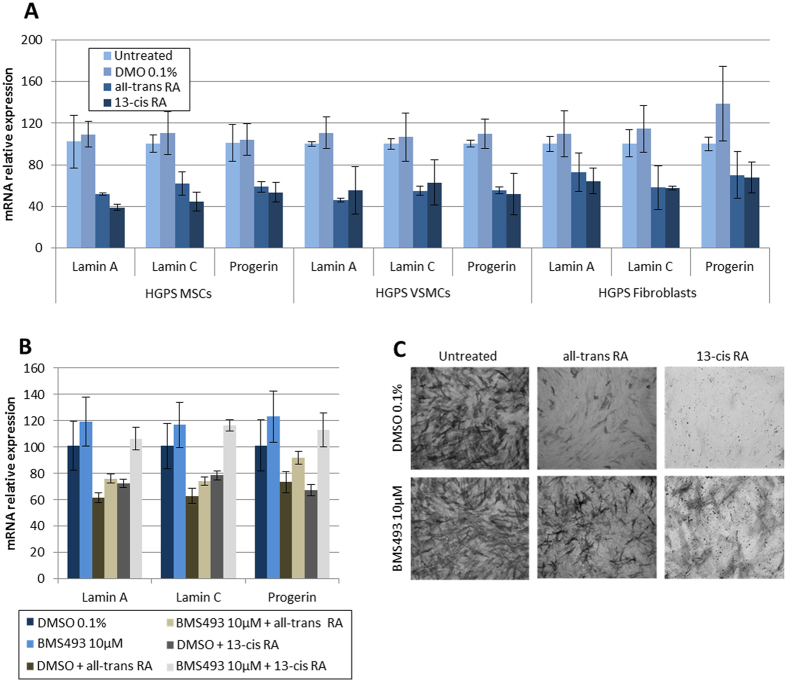
Regulation of osteogenic differentiation in HGPS OP by retinoic acids. (**A**) Quantification of effect of retinoic acids (10 μM) on lamin A, lamin C, progerin mRNA expression in HGPS MSCs, HGPS vascular smooth muscle cells (VSMCs) and primary HGPS fibroblasts. (**B**) Gene expression analysis for lamin A, lamin C, progerin in HGPS MSCs following 72 hours of treatment with the retinoic acid receptor antagonist BMS493 (10 μM) in combination with, or without, all-trans or 13-cis-RA (10 μM). (**C**) Alkaline phosphatase activity in HGPS osteogenic progenitors following 7 days of differentiation in the presence of BMS493 (10 μM) in combination with, or without, all-trans RA or 13-cis-RA. Datas are presented as a percentage relative to untreated cells for each cell types.

To validate the rationale of our screening cascade and because secondary assays revealed that retinoids were the only compounds capable to efficiently act on progerin expression, the last part of this study was focused on the characterization of RAs molecular mechanisms. First, 13-cis RA and all-trans RA effects on lamins expression were confirmed at the protein level in HGPS MSCs by western blot (Fig. 4C). Dose-response curves were established using the same cellular model, showing an effect on lamin expression in the nanomolar range (100 nM) (Fig. 4D,E). Finally, effects of all-trans RA and 13-cis RA on progerin, lamin A and lamin C expression were confirmed by qPCR in other cell types, i.e. primary fibroblasts and vascular smooth muscle cells (VSMCs) derived from HGPS iPS cells (Fig. 5A). Because RARE (retinoic acid responsive elements) motifs are also present in the *LMNA* promoter^26^, their involvement in the molecular mechanisms driving the effects of retinoids on progerin expression was evaluated using BMS493, an RAR antagonist. Accordingly, HGPS MSCs were treated for 48 h with 10 μM BMS493, in the absence of RAs. Measurement of A-type lamin expression revealed an increase in lamin A, lamin C and progerin mRNA expression in presence of the inhibitor (Fig. 5B). In contrast, when treated in the presence of RAs, 10 μM BMS493 strongly inhibited LMNA repression mediated by 13-cis RA and all-trans RA (Fig. 5B). Finally, these results were confirmed, showing that BMS493 also strongly reduced the effects of RAs on the osteogenic differentiation of HGPS MSCs (Fig. 5C).

## Discussion

The main result of this study is the demonstration that iPSC derivatives can be used to identify drugs that normalize pathological phenotypes associated with HGPS. This was accomplished through unbiased high-throughput screening of 2800 compounds, highlighting the therapeutic potential of retinoids for the treatment of HGPS. This result strongly underscores the value of such models for pharmacological approaches to monogenic diseases.

### High-throughput drug screening on HGPS cells

High-throughput screening is a drug-discovery process that is widely used in order to rapidly assess a large number of candidate compounds in a standardized and reproducible manner. In the context of HGPS, two research groups have recently reported screenings of large chemical libraries. One of these groups was searching for inhibitors of prelamin A maturation^20^ and the other for compounds that rescue pathological defects associated with progerin overexpression^19^. In this paper, we describe an alternative method that measures alkaline phosphatase activity in HGPS MSCs committed to the osteogenic differentiation process. This phenotype was initially described in healthy MSCs that overexpressed progerin^6^ and later confirmed in MSCs derived from HGPS iPS, with the increased alkaline phosphatase activity being normalized by the different drugs used on HGPS patients, FTIs, ZoPra and rapamycin^7^. This phenotype was subsequently used to characterize the functional effect of ten new inhibitors of prelamin A farnesylation^20^, with all these compounds also rescuing this phenotype. Here, we reinforce the pathological relevance of this readout by reporting that known inhibitors of osteogenesis have no effect on alkaline phosphatase activity in HGPS MSCs committed to the osteogenic differentiation process, suggesting the involvement of HGPS-specific molecular mechanisms in the control of this process of differentiation. Conversely, functional characterization of the compounds identified in our screening revealed that seven of the ten hits were effectively inhibitors of the osteogenic differentiation process.

### Identification of seven regulators of premature osteogenic differentiation in HGPS cells

Measurement of progerin expression and prelamin A maturation revealed that, among this list of seven compounds, a PI3K inhibitor (LY-293002), a dihydrofolate reductase inhibitor (methotrexate) and a selective serotonin releasing agent (PMA) partially inhibited prelamin A maturation. Even if the effects of these drugs on prelamin A are only partial (less than 25% of inhibition), further experiments should be performed to evaluate potential interactions for these three pathways and the prenylation pathway. Our screening process also identified two compounds that normalize osteogenic differentiation, with no effect on progerin expression or prelamin A, a purine analogue (azathioprine) and a 5-HT2C receptor antagonist (SB-242.084). The fact that no other antimetabolites and 5-HT2C receptor antagonists were positive in that screening suggests that these two compounds are both acting through an off-target effect that remains to be identified. However, these results are line with the recent report describing that compounds can normalize some progeroid phenotypes through a progerin-independent mechanism, as described for the inhibition of the acetyl-transferase protein, NAT10^27^. Finally, secondary assays revealed that among these seven hits, two physiologically active metabolites of vitamin A, all-trans RA (also known as tretinoin), and one of its derivatives, 13-cis RA, were capable of suppressing progerin expression above 30%, with no toxic effects. RAs have been reported to regulate various biological processes, including cell proliferation and differentiation processes, through activation of retinoic acid receptors (RAR) and retinoid X receptors (RXR)^28^. RA receptors can act on gene expression either as suppressors or as activators of transcription, depending on the cofactors that are associated with the complex^29^. In 2012, Swift *et al.* reported that RA acted as a suppressor of LMNA transcription in healthy cells through the direct binding of RAR to the RAR elements located within LMNA promoter^26^. Our results show the very same effect of RA and RAR antagonists on LMNA transcription in HGPS cells, confirming that measurement of osteogenic differentiation in HGPS MSCs can allow the identification of progerin regulators. Beyond the identification of RAs, taken together, these results suggest that it would be possible to identify other new progerin regulators by screening a larger compound library on this readout.

### Retinoids regulate premature osteogenic differentiation in HGPS cells

In recent years, several studies have described RAs as positive regulators of osteogenic differentiation of MSCs in non-pathological contexts^30^. In contrast, we report here that RAs act as suppressors of premature osteogenic differentiation. This intriguing peculiarity to the HGPS model suggests that specific RA-independent molecular mechanisms are involved in this pathological differentiation process, or that specific molecular mechanisms associated with HGPS cells impact on the response of MSCs to RAs. In both cases, identification of these potential pathways might facilitate the identification of new potential pharmacological targets for HGPS. Recently, RAs were reported to regulate osteogenic differentiation of MSCs through the direct binding of RAR/RXR to a retinoic acid responsive element within the *LMNA* promoter^26^. The role of RA-mediated lamin regulation was subsequently reinforced by showing that lamin A/C knockdown inhibits retinoic acid-mediated differentiation of human neuroblastoma cells^31^. Taken together, these two studies report that RAs regulate lamin A/C expression to control some of the RA-mediated biological processes by opening up a set of new applications for other lamin A/C regulators. In 2015, two groups used this property to investigate the effect of RAs on progerin expression in HGPS skin fibroblasts^16^,^19^. This was first demonstrated using high-content screening performed on healthy fibroblasts that overexpressed progerin. Among the hits identified, the retinoids were the most efficient at decreasing progerin expression and improving nuclear shape architecture (LAP2-gamma, lamin B1), the DNA repair process (H2AX, 53BP1) and H3K27 trimethylation^19^. Our results are in line with these studies, confirming the link between the RA pathway and progerin expression and, in addition, describing their effect on the premature differentiation process.

### Retinoid potential for clinical applications

All-trans RA is commonly used in dermatology to treat acne vulgaris and keratosis pilaris^32^ due to its role in the regulation of keratinocyte differentiation^33^. In the past 30 years, because of their reported effects on colony formation and transformation in malignant cells^34^,^35^, RAs have also been investigated as therapeutic agents for a variety of cancers^36^, with significant activity described for the treatment of promyelocytic leukemia^37^,^38^,^39^,^40^. Even if further animal studies in relevant animal models for HGPS are required before any application of RAs can be envisaged in HGPS patients, our results highlight that this therapeutic option may merit further study. RA treatment can be associated with drugs that elicit degradation of progerin in order to enhance the progerin decrease in HGPS cells, as has recently been described^16^. However, no evidence has been reported regarding its association with FTIs. FTIs, such as ionafarnib, decrease prelamin A farnesylation and improve pathological defects associated with HGPS *in vitro* and *in vivo*. However, it has been reported that accumulation of unfarnesylated prelamin A is associated with a certain degree of toxicity *in vitro*^7^ and with cardiomyopathy *in vivo*^41^. Taking into account the fact that RAs regulate prelamin A transcription, these compounds could also theoretically be used to decrease FTI-mediated toxicity by limiting the accumulation of non-farnesylated prelamin A. Further preclinical evaluations of cross-combinations of RAs and FTIs should be carried out in relevant animal models for HGPS to define the optimum subtoxic dose for these two types of treatments. Finally, beyond their application in HGPS, the effect of RAs on lamin A and lamin C also highlights the potential use of these compounds as treatment for hundreds of other diseases caused by other mutations in the *LMNA* gene, grouped under the term of laminopathies.

## Material and Methods

### Fibroblast culture and reprogramming

HGPS fibroblasts (13–8243) used in this study were isolated from a patient biopsy taken in the Assistance Publique Hôpitaux de Marseille. Informed consents were obtained from the parents of the patient included in this study, complying with the ethical guidelines of the institutions involved and with the legislation requirements of the country of origin. The HGPS cell line explored in this study has been prepared and stored according to the French regulation by the Biological Resource Center of Tissues, DNA, Cells (CRB TAC), Department of Medical Genetics, la Timone Hospital, Marseille. Experimental protocols were approved by the french minister of health: DC-2008-429. The control cell line was provided by Coriell Cell Repository (Camden, USA). Cells were obtained from the NINDS Human Genetics Resource Center DNA and Cell Line Repository. NINDS Repository sample numbers corresponding to the samples used are DM4603. Cultures were maintained in Dulbecco’s modified Eagle’s medium + GlutaMAX II + 4500 mg/L D-Glucose (Gibco), supplemented with 20% fetal bovine serum (research grade, Sigma) and 1% sodium pyruvate 100 mM (Life technologies). Cell cultures were maintained at 37 °C, in 5% CO_2_ in a humidified atmosphere, with the media changed every 2 days. Fibroblasts from 13–8243 and DM4603 were reprogrammed into iPS cells using Yamanaka’s original method with OCT4, KLF4, SOX2, c-myc, and transferred using retroviral vectors^42^.

### Pluripotent stem cell culture and differentiation

WT and HGPS iPSCs were grown in colonies on mouse embryonic fibroblasts (MEF), inactivated with 10 mg/ml mitomycin C seeded at 30,000 cells/cm^2^, and grown as previously described. iPSC were differentiated into mesenchymal stem cells (MSCs) using directed protocols for differentiation previously published by our group^24^. SMC differentiation of iPSC was performed using embryoid body (EB) formation, as previously described^43^,^44^. EBs were cultured for 10 days at 37 °C, in 5% CO_2_ in a humidified atmosphere, with media changes every 2 days. CD34+ cells were isolated from EBs on day 10. The percentage of CD34+ cells in EBs was between 0.4 and 1.5%. Isolated cells were grown on 24-well plates (~30,000 cells/cm^2^) coated with 0.1% gelatin, in the presence of endothelial growth medium-2 (EGM-2, Lonza) supplemented with PDGFBB (50 ng/mL, Prepotech). After 4 passages, the medium was replaced with Smooth Muscle Growth Medium-2 (SmGM-2) (Lonza CC-3182) for 4 additional passages. Cell cultures were maintained at 37 °C, in 5% CO_2_ in a humidified atmosphere, with the media changed every 2 days.

### Cell culture and drug treatments

MSCs derived from HGPS iPSCs (HGPS MSCs) and WT iPSCs (WT MSCs) were cultured in KnockOut Dulbecco’s modified Eagle’s medium (Invitrogen, Carlsbad, CA), supplemented with 20% fetal bovine serum (research grade, Sigma), 1% non-essential amino acids (Invitrogen), 1% glutamax (Invitrogen), and 0.1% β-mercaptoethanol (Invitrogen). Cell cultures were maintained at 37 °C, in 5% CO_2_ in a humidified atmosphere, with the media changed every 2 days. Six hours after seeding, MSCs were treated with 0.1% dimethyl sulfoxide, 3 μM FTI (tipifarnib, R115777; Selleck Chemicals, Houston, TX), 1 μM methoctramine tetrahydrochloride (M105, Sigma), 10 μM all-trans retinoic acid (R2625, Sigma), 0.1 μM phorbol 12-myristate 13-acetate (1201, Tocris), 25 μM LY-294.002 hydrochloride (sc-215273A, Santa Cruz), 12.5 μM 8-Bromo-cAMP sodium (B7880, Sigma), 10 μM 13-cis-retinoic acid (5513, Tocris), 10 μM methotrexate (A6770, Sigma), 10 μM azathioprine (A4638, Sigma), 50 μM SB 242.084 dihydrochloride hydrate (2901, Tocris), 50 μM SMER28 (4297, Tocris). Cells were analyzed after 72 hours of treatment.

### Osteogenic differentiation

Cells were seeded at 12,000 cells for WT MSC and 19,000 cells for HGPS MSCs per well in 24-well plates in MSCs culture medium and treated as previously described. After 72 hours, the MSC medium was replaced with STEMPRO osteogenic induction medium (Invitrogen), in the presence of or without the different drugs. After 7 days of treatment, the cells were fixed with 95% ethanol and stained by adding either a colorimetric substrate for alkaline phosphatase, 5-bromo-4-chloro-3-indolyl phosphate/nitro blue tetrazolium (NBT) (Sigma-Aldrich), or a chromogenic substrate for this enzyme (absorbance at 405 nm), p-nitrophenylphosphate (pNPP) (Pierce Biotechnology). Cell viability was measured by counting Hoechst stained cells using a LEAP cell processing workstation (Cyntellect). Data are expressed as percentages relative to the control values which are defined as 100%.

### Quantitative PCR

Total RNA was isolated using an RNeasy Micro extraction kit (Qiagen, Courtaboeuf, France), according to the manufacturer’s protocol. An on-column DNase I digestion was performed to avoid genomic DNA amplification. RNA level and quality were checked using the Nanodrop technology. A total of 500 ng of RNA was used for reverse transcription using the Superscript III reverse transcription kit (Invitrogen). Q-PCR analysis was performed using an ABI 7900 system or a QuantStudio 12 K Flex real-time PCR system (Applied biosystem) and TaqMan gene expression Master Mix (Roche) or Luminaris Probe qPCR Master Mix (Thermo Scientific), respectively, following the manufacturers’ instructions. Quantification of gene expression was based on the DeltaCt Method and normalized to 18S expression. PCR primers were previously described by S. Rodriguez and colleagues^45^. Primer sequences were lamin A (exons 11/12), 5′-TCTTCTGCCTCCAGTGTCACG-3′ and 5′-AGTTCTGGGGGCTCTGGGT-3′; lamin C (exons 9/10), 5′-CAACTCCACTGGGGAAGAAGTG-3′ and 5′-CGGCGGCTACCACTCAC-3′ and progerin (exons 11/12), 5′-ACTGCAGCAGCTCGGGG-3′ and 5′-TCTGGGGGCTCTGGGC-3′; ALPL, 5′-CCACGTCTTCACATTTGGTG-3′ and 5′-AGACTGCGCCTGGTAGTTGT-3′; COL1A1, 5′-CCCCTGGAAAGAATGGAGAT-3′ and 5′-CCATCCAAACCACTGAAACC-3′; OCN, 5′-GCTGAGTCCTGAGCAGCAG-3′ and 5′-CCAATAGGGCGAGGAGTGTG-3′. Taqman MGB probe sequences were lamin A (exon 11), 5′-ACTCGCAGCTACCG-3′; lamin C (exon 10), 5′-ATGCGCAAGCTGGTG-3′ and progerin (exon 11), 5′-CGCTGAGTACAACCT-3′. Reporter and quencher dyes for the LMNA locus assays were 5′ 6FAM and 3′- non-fluorescent quencher dye (NFQ; Applied Biosystems). 18 s (Assay HS_99999901_s1) probes and primers were provided by life technologies.

### Western immunoblotting

Whole-cell lysates of MSCs were collected, proteins were extracted in NP40 cell lysis buffer (Invitrogen) with a protease and phosphatase inhibitor cocktail (ThermoScientific). Lysates were sonicated 4 times for 15 sec each, each with an interval of 15 sec in between, and then centrifuged at 10,000 g for 10 minutes at 4 °C. Protein concentration was measured using the Pierce BCA Protein Assay Kit (ThermoScientific) and the absorbance at 562 nm was evaluated using a Clariostar (BMG Labtech). A total of 20 μg of protein was loaded and run on a 7% tris-acetate gel (Criterion™ XT) using XT tricine running buffer (Biorad). Gels were then transferred onto polyvinylidene fluoride (PVDF) membranes (Biorad) using a Trans-Blot Turbo Transfer System (Biorad). Blots were blocked in Rockland blocking buffer (Millipore) diluted 1:2 in TBS 1x for 1 hour at room temperature. Membranes were incubated with primary antibodies diluted in blocking buffer with 0.1% Tween20 (VWR) overnight at 4 °C. The primary antibodies used here are a mouse anti-lamin A/C 1:200 (Millipore, JOL2, MAB3211), a mouse anti-progerin 1:200 (SantaCruz, 13A4D4, sc-81611) and a mouse anti-actin 1/5,000 (Millipore, MAB1501R). Washing was carried out for 45 minutes at room temperature with TBS + 0.1% Tween20 and the membranes were incubated with an IR-Dye 800CW conjugated with a secondary donkey anti-mouse antibody or a IR-Dye 680RD conjugated with a secondary donkey anti-mouse antibody (LI-COR Biosciences) at 1:10,000 in blocking buffer with 0.1% Tween20 and 0.01% SDS (Ambion). The detector was an Odyssey Infrared Imaging System (LI-COR Biosciences).

### Immunocytochemistry

Cells were fixed in 4% paraformaldehyde (15 minutes, room temperature) before permeabilization in PBS supplemented with 0.1% triton X-100 (Sigma) (5 minutes, room temperature). They were then blocked during 30 minutes at room temperature using PBS with 1% BSA (Sigma-Aldrich, St. Louis, USA). The primary antibody, rabbit anti-prelamin A (ANTOO45, Diatheva), was incubated for one hour at room temperature in blocking buffer. Cells were stained with the species-specific fluorophore-conjugated secondary antibody (Invitrogen) (one hour, room temperature) and nuclei were visualized with Hoechst 33342 (Invitrogen). The percentage of prelamin A positive nuclei was analyzed using an ArrayScan VTI HCS Reader (Cellomics).

### Statistical analysis

Statistical analysis was performed with one-way analysis of variance (ANOVA), using Dunnet’s comparison test. Values of p < 0.05 were considered significant (*p < 0.05, **p < 0.01, ***p < 0.001).

## Additional Information

**How to cite this article**: Lo Cicero A. *et al.* A High Throughput Phenotypic Screening reveals compounds that counteract premature osteogenic differentiation of HGPS iPS-derived mesenchymal stem cells. *Sci. Rep.* **6**, 34798; doi: 10.1038/srep34798 (2016).

## Supplementary Material

Supplementary Information
