# Supplementary material for: A High Throughput Phenotypic Screening reveals compounds that counteract premature osteogenic differentiation of HGPS iPS-derived mesenchymal stem cells
Source: Sci Rep. 2016 Oct 14;6:34798. doi: 10.1038/srep34798 (PMC5064407; doi:10.1038/srep34798)
Supplement: Supplementary Information [file srep34798-s1.pdf]

Supplementary information

A High Throughput Phenotypic Screening reveals compounds that counteract premature osteogenic differentiation of HGPS iPS-derived mesenchymal stem cells

Alessandra LO CICERO and Anne-Laure JASKOWIAK, Anne-Laure EGESIPE , Johana TOURNOIS, Benjamin BRINON, Patricia R PITREZ, Lino FERREIRA, Annachiara DE SANDRE-GIOVANNOLI, Nicolas LEVY, Xavier NISSAN

Sup Figure 1

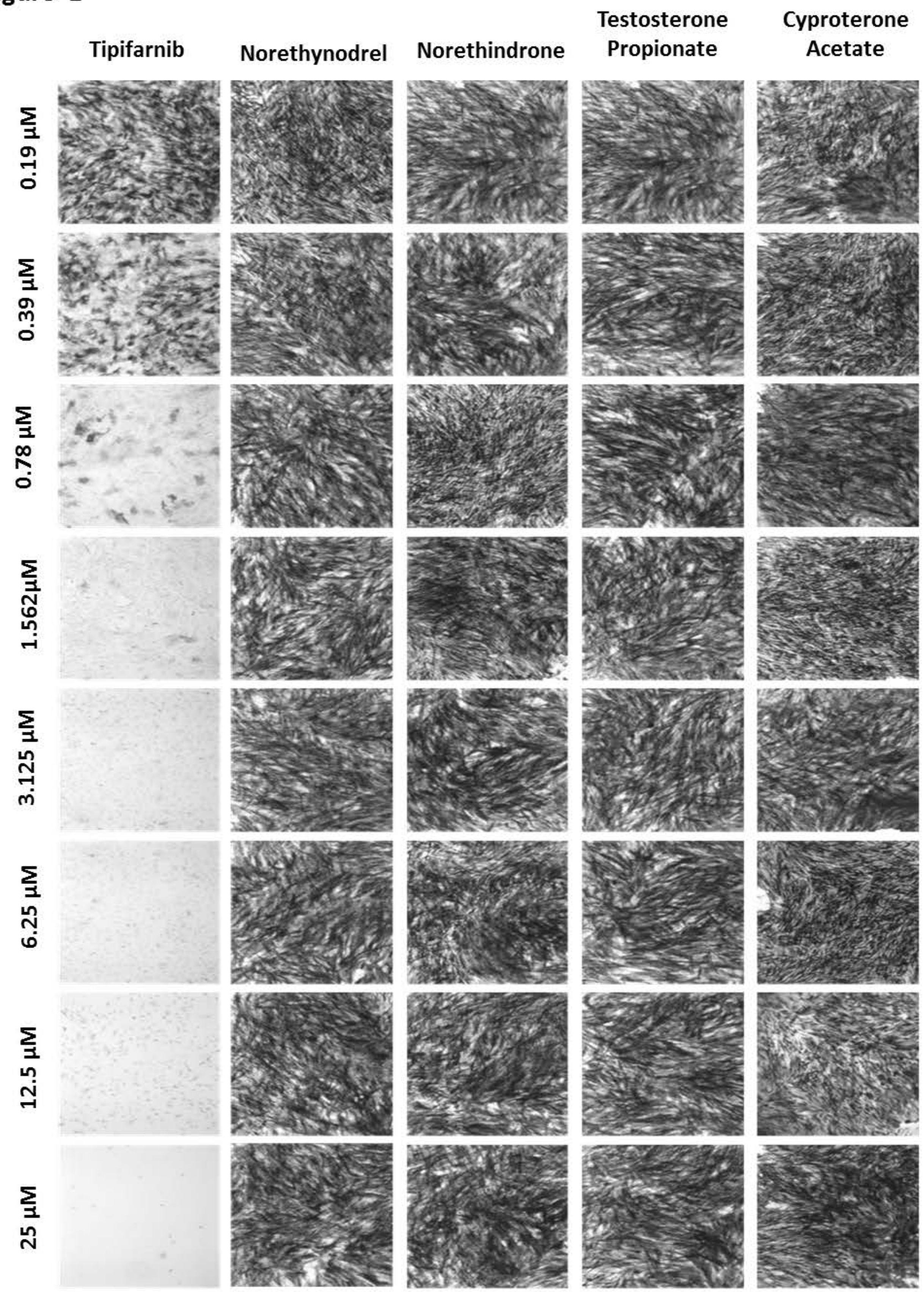

Sup Figure 1: Effect of known inhibitors of osteogenesis on HGPS MSCs premature osteogenic differentiation in MSCs.

Alkaline phosphatase activity in HGPS osteogenic progenitors following 7 days of differentiation in the presence of different doses of osteogenesis regulators.

Sup Figure 2

A

	Nb compounds	Concentration	Chemical diversity
Prestwick chemical library 1	1120	0.2µM	- FDA approved drugs<br>- Interesting for a drug repositioning approach
Prestwick chemical library 2	400	2µM-10µM	- Small molecules that belong to the Prestwick Pyridazine library.
LOPAC chemical library	1280	10µM	- Pharmacologically active compounds<br>- Use to identify biological pathways

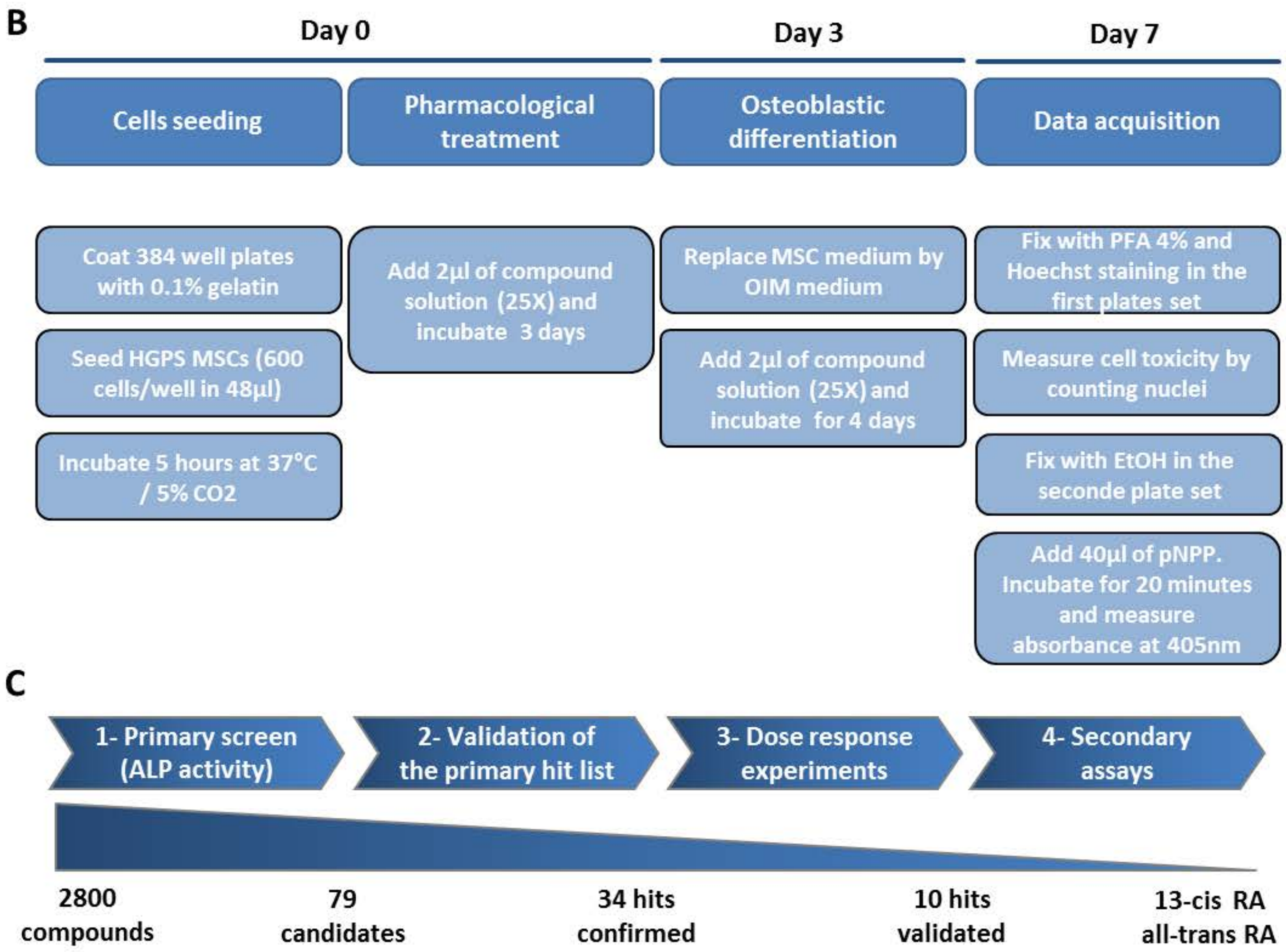

Sup Figure 2: Experimental workflow for the screening procedure to detect inhibitors of HGPS MSCs premature osteogenic differentiation.

(A) Composition of the different chemical compound libraries used in the screening.

(B) Main steps of the screening procedure.

(C) Schematic representation of the 4-step analysis leading to the identification of the hit list.

Sup Figure 3

Name	Abbreviation	Library	Dose	Screening		Retest		IC50	Dose
				ALP (%)	Viability (%)	ALP (%)	Viability (%)		
Methoctramine tetrahydrochloride	Methoctramine	Lopac	10µM	5.64	60.83	4.73	58.16	0.8µM	1µM
all-trans retinoic acid	all-trans RA	Lopac	10µM	6.38	68.78	7.76	77.32	2.5µM	10µM
Phorbol 12-myristate 13-acetate	PMA	Lopac	10µM	7.61	90.54	7.51	93.4	25nM	100nM
LY-294.002 hydrochloride	LY-294.002	Lopac	10µM	7.3	86.09	5.79	89.63	7.7µM	25µM
8-Bromo-cAMP sodium	8-Bromo-cAMP	Lopac	10µM	9.24	65.56	12.92	59.3	6.3µM	12.5µM
13-cis retinoic acid	13-cis RA	Lopac	10µM	10.95	81.63	18.49	81.11	4.8µM	10µM
Methotrexate	Methotrexate	Prestwick	0.2µM	18.09	48.82	31.92	61.06	95nM	10µM
Azathioprine	Aza	Prestwick	0.2µM	20.79	113.03	20.74	100.83	4µM	10µM
SB 242.084 dihydrochloride hydrate	SB-242.084	Lopac	10µM	27.75	111.05	30.03	108.08	31µM	50µM
SMER28	SMER28	Lopac	10µM	28.92	137.15	23.09	138.66	19µM	50µM

Sup Figure 3: List of the ten hits identified in this screening

List of the 10 modulators identified after screening validation. The IC50 value corresponds to the dose that induces a 50% decrease in alkaline phosphatase activity. The dose in the final column corresponds to the dose used for the molecular characterization and secondary assays. Data are expressed as percentages relative to the control values which are defined as 100%.

Sup Figure 4

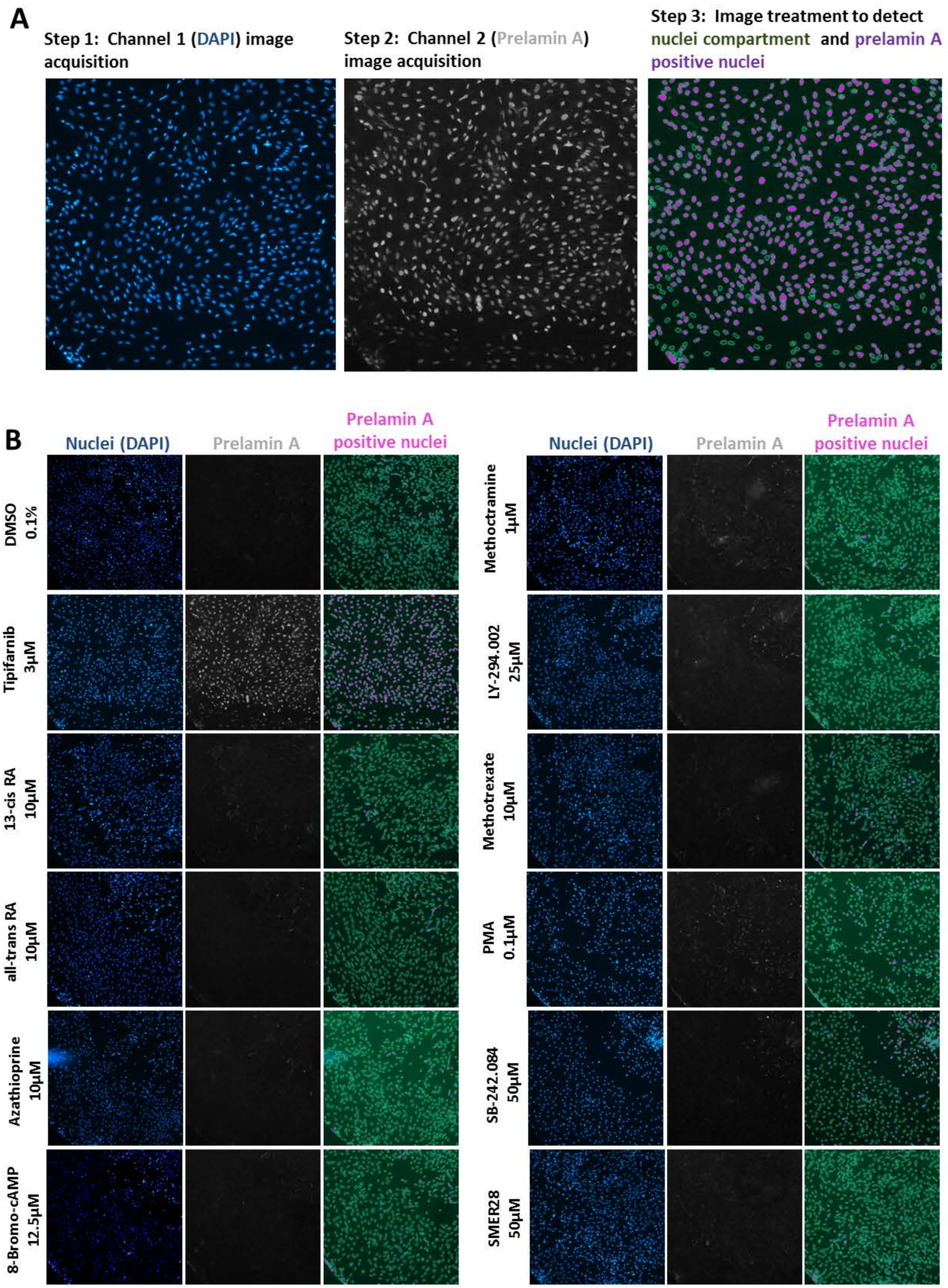

Sup Figure 4: Measure of prelamin A maturation in HGPS MSCs

(A) 3 steps-workflow to measure the percentage of prelamin A positive nuclei in HGPS MSCs. First step consist to detect nuclei compartement (channel 1 / DAPI staining). Then, prelamin A signal (channel 2 / Prelamin A) is quantified using a specific antibody targeting prelamin A and not mature forms of A types lamins. Finally our algorithm detect nuclei compartement (colored in green) and calculate the percentage of nuclei presenting prelamin A staining (colored in purple).

(B) Analysis of premain A maturation following 48h with the ten hits identified in this screening. Tipifarnib 3µM was used as positive control.

Sup Figure 5

	Primary readout (ALP activity)	Osteogenic differentiation	Prelamin A maturation	Progerin expression
All-trans RA	✓	✓	✗	✓
13-cis RA	✓	✓	✗	✓
LY-294.002	✓	✓	✓	✗
Methotrexate	✓	✓	✓	✗
PMA	✓	✓	✓	✗
Azathioprine	✓	✓	✗	✗
SB.242.084	✓	✓	✗	✗
8-Bromo-cAMP	✓	✗		
Methoctramine	✓	✗		
SMER28	✓	✗		

Sup Figure 5: Results of the screening

Measure of ALP activity in HGPS osteogenic progenitors revealed 10 hits. Among them, three were considered as false positive hits since they do not modulate osteogenic genes (8-Bromo-cAMP, Methoctramine and SMER28). Functional secondary assays revealed that two of these hits were repressing progerin expression (all-trans RA and 13-cis RA), three of them were slightly acting on prelamin A maturation (LY-294.002, Methotrexate and PMA) and two compounds were not acting on any of these parameters (Azathioprine and SB.242.084)

Sup Table 1

	SAMPLE_NAME	SAMPLE_ALIAS	NAME	DOSE	Primary screening		Retest	
					Viability_%	ALP_%	Viability_%	ALP_%
**Selective Serotonin Reuptake Inhibitor (SSRI) or Norepinephrine Reuptake Inhibitor**								
	C000108-B	Prestw-108	Nomifensine maleate	0.2µM	100.96	26.8	Negative	
	C000511-D	510(CATNUM:F-132)	Fluoxetine hydrochloride	10µM	35.38	8.09	66.79	5.69
	C284034-A	1072(CATNUM:M 9651)	Maprotiline hydrochloride	10µM	46.48	5.9	41.06	5.35
	C000254-D	524(CATNUM:N 7261)	Nortriptyline hydrochloride	10µM	61.37	11.72	52.88	5.81
	C000445-D	773(CATNUM:C 4542)	Cyclobenzaprine hydrochloride	10µM	92.47	12.39	Negative	
	C284027-A	521(CATNUM:M-149)	Methiothepin mesylate	10µM	75.88	18.79	Negative	
	C283841-A	908(CATNUM:F 1553)	S-(+)-Fluoxetine hydrochloride	10µM	79.17	26.58	75.76	8.63
	C284108-A	58(CATNUM:S 8061)	SB 242084 dihydrochloride hydrate	10µM	111.05	27.75	108.08	30.03
	C284112-A	1124(CATNUM:L9793)	LP44	10µM	89.27	28.92	Negative	
	C000930-D	146(CATNUM:P 8813)	Protriptyline hydrochloride	10µM	83.27	29	72.96	32.28
**Kinase inhibitor**								
	C283542-A	126(CATNUM:I0404)	Indirubin-3'-oxime	10µM	75.82	5.92	Negative	
	C283955-A	1349(CATNUM:L 9908)	LY-294.002 hydrochloride	10µM	86.09	7.3	89.63	5.79
	C283572-A	415(CATNUM:B 7880)	8-Bromo-cAMP sodium	10µM	65.56	9.24	59.3	12.92
	C283915-A	998(CATNUM:K 3888)	Kenpaullone	10µM	88.23	15.99	Negative	
	C283790-A	884(CATNUM:S 3567)	SB 415286	10µM	69.02	19.18	85.35	28.09
	C284182-A	1158(CATNUM:P 8139)	Phorbol 12-myristate 13-acetate	10µM	90.54	7.61	93.4	7.51
	C284257-A	1247(CATNUM:T 4182)	Tyrphostin AG 1478	10µM	31.13	11.52	88.94	6.56
	C284333-A	1270(CATNUM:T 7665)	Tyrphostin 51	10µM	56.47	14.03	Negative	
	C283784-A	862(CATNUM:S 5567)	SP600125	10µM	85.52	31.29	Negative	
	C284120-A	1136(CATNUM:N 4286)	NU2058	10µM	74.59	32.76	Negative	
	C284318-A	1275(CATNUM:N 4411)	NU6027	10µM	66.06	22.28	Negative	
**Dopamine Receptor**								
	C000097-B	Prestw-97	Disulfiram	0.2µM	74.34	4.74	Negative	
	C283485-A	1342(CATNUM:A 4393)	Apomorphine hydrochloride hemihydrate	10µM	80.41	5.38	Negative	
	C283979-A	1023(CATNUM:L-135)	L-741.626	10µM	65.8	5.82	48.53	4.95
	C283763-A	872(CATNUM:D5676)	N-Desmethylclozapine	10µM	88.69	19.11	77.81	23.59
	C283769-A	267(CATNUM:D-030)	R(-)-2.10.11-Trihydroxy-N-propylnoraporphine hydrobromide	10µM	84.81	22.9	Negative	
	C283752-A	265(CATNUM:D-027)	R(-)-Propylnorapomorphine hydrochloride	10µM	73.76	26.03	Negative	
	C284155-A	1155(CATNUM:P 7780)	Propionylpromazine hydrochloride	10µM	56.06	11.22	Negative	
	C000364-D	522(CATNUM:M 3668)	Metergoline	10µM	80.49	30.77	Negative	
**Histamine Inhibitor**								
	C283692-A	180(CATNUM:E9531)	Ebastine	10µM	86.87	6.64	Negative	
	C283875-A	437(CATNUM:M 7320)	N-Methylhistaprodifen dioxalate salt	10µM	38.83	6.64	35.68	5.25
	C283622-A	805(CATNUM:C 8903)	Clemastine fumarate	10µM	34.7	5.72	85.26	11.26
	C284343-A	477(CATNUM:S 5317)	SKF 95282 dimaleate	10µM	78.93	14.93	Negative	
**Metabolism**								
	C000135-B	Prestw-135	Methotrexate	0.2µM	48.82	18.09	61.06	31.92
	C283448-A	485(CATNUM:A 4638)	Azathioprine	0.2µM	113.03	20.79	100.83	20.74
	C000373-B	Prestw-373	Amethopterin (R.S)	0.2µM	48.06	30.78	Negative	
	C283442-A	676(CATNUM:A 6770)	Methotrexate hydrate	10µM	53.64	13.41	53.59	25.06
	C283454-A	621(CATNUM:A 1784)	Aminopterin	10µM	58.51	14.56	56.01	27.2
	C283595-A	721(CATNUM:B 5002)	5-Bromo-2'-deoxyuridine	10µM	31.87	30.84	Negative	
**Retinoic Acid**								
	C000257-D	1183(CATNUM:R 2625)	Retinoic acid	10µM	68.78	6.38	77.32	7.76
	C284295-A	1244(CATNUM:T 3757)	TTNPB	10µM	72.82	10.43	83.22	8.55
	C000256-D	1185(CATNUM:R 3255)	13-cis-retinoic acid	10µM	81.63	10.95	81.11	18.49
	C283895-A	957(CATNUM:H 7779)	Retinoic acid p-hydroxyanilide	10µM	81.07	26.69	71.55	7.07
**Cardiac Glycoside**								
	C000656-B	Prestw-656	Lanatoside C	0.2µM	107.61	7.38	Negative	
	C000436-B	Prestw-436	Digitoxigenin	0.2µM	53.54	4.96	42.02	6.33
	C000437-B	Prestw-437	Digoxin	0.2µM	35.98	7.19	39.53	5.84
**Adrenergic receptor inhibitor**								
	C283687-A	108(CATNUM:C-223)	Cirazoline hydrochloride	10µM	49.39	11.45	Negative	
	C284260-A	1226(CATNUM:S 8688)	SR 59230A oxalate	10µM	45.52	6.64	Negative	
	C284236-A	1224(CATNUM:S 5068)	Salmeterol xinafoate	10µM	49.2	18.64	56.27	5.73
**Diuretic**								
	C283460-A	665(CATNUM:A 3085)	5-(N-Ethyl-N-isopropyl)amiloride	10µM	35.68	4.43	42.96	5.1
	C284210-A	71(CATNUM:S 3378)	Spironolactone	10µM	42.25	13.35	46.53	6.4
	C000657-D	713(CATNUM:B 2417)	Benzamil hydrochloride	10µM	100.66	32.63	Negative	
**Other**								
	C001082-B	Prestw-1082	(-)-Eseroline fumarate salt	10µM	56.7	20.74	Negative	
	C293166-A	PCIPYR-0057		0.0047	90.55	23.24	Negative	
	C293191-A	PCIPYR-0084		0.0065	167.13	29.85	Negative	
	C293339-A	PCIPYR-0289		0.0051	75.22	29.81	Negative	
	C283510-A	1353(CATNUM:PZ0015)	Trovafloxacin mesylate	10µM	31.34	5.35	Negative	
	C284072-A	206(CATNUM:M-105)	Methoctramine tetrahydrochloride	10µM	60.83	5.64	58.16	4.73
	C283814-A	911(CATNUM:F 3764)	Furegrelate sodium	10µM	37.03	9.88	Negative	
	C283749-A	879(CATNUM:E 1779)	ET-18-OCH3	10µM	36.19	12.32	Negative	
	C000308-D	296(CATNUM:P 1793)	Pimozide	10µM	85.36	19.27	Negative	
	C283576-A	744(CATNUM:C 0400)	Carmustine	10µM	51.92	22.48	62.44	17.81
	C283698-A	837(CATNUM:D 5294)	Dilazep hydrochloride	10µM	71.65	25.41	Negative	
	C283884-A	937(CATNUM:J4829)	JFD00244	10µM	42.09	25.85	97.11	6.3
	C000146-D	466(CATNUM:T 9262)	Tamoxifen citrate	10µM	41.13	12.23	Negative	
	C283477-A	656(CATNUM:A 4147)	3-Amino-1-propanesulfonic acid sodium	10µM	114.72	28.27	115.23	23.53
	C000993-D	1194(CATNUM:R 9644)	Ribavirin	10µM	63.88	24.49	69.9	22.59
	C284206-A	397(CATNUM:R-116)	Riluzole	10µM	51.51	26.56	Negative	
	C284173-A	541(CATNUM:S 8197)	SMER28	10µM	137.15	28.92	138.66	23.09
	C000267-D	785(CATNUM:C 6019)	Clotrimazole	10µM	69.52	30.6	Negative	
	C000790-B	Prestw-790	Cycloheximide	0.2µM	91.91	33.96	Negative	
	C283656-A	70(CATNUM:C 4238)	CNS-1102	10µM	95.52	30.03	Negative	
	C283712-A	825(CATNUM:D 1916)	D-ribofuranosylbenzimidazole (DRB)	10µM	59.29	30.74	Negative	
	C283743-A	858(CATNUM:D 8296)	3-deazaadenosine	10µM	96.71	31.29	Negative	
	C283801-A	905(CATNUM:T 5648)	Tamoxifen	10µM	80.88	30.37	Negative	
	C284040-A	1098(CATNUM:N 8659)	Nimustine hydrochloride	10µM	52.25	30.25	Negative	
	C293151-A	PCIPYR-0041		0.0065µM	32.94	31.27	Negative	
	C293371-A	PCIPYR-0324		0.0062µM	68.34	33.9	Negative	
	C293195-A	PCIPYR-0088		0.0062µM	119.05	34.08	Negative	

Sup Table 1: Results of the screening

Classified list of the primary hits identify during the screening. Results of the primary test and retest on Alkaline phosphatase activity and cell viability
